# Energy allocation shifts from sperm production to self-maintenance at low temperatures in male bats

**DOI:** 10.1038/s41598-022-05896-3

**Published:** 2022-02-08

**Authors:** Ewa Komar, Nicolas J. Fasel, Paulina A. Szafrańska, D. K. N. Dechmann, Marcin Zegarek, Ireneusz Ruczyński

**Affiliations:** 1 Mammal Research Institute, Polish Academy of Sciences, Stoczek 1, Białowieża, Poland; 2 Faculty of Biology, University of Warsaw, Miecznikowa 1, 02-096 Warszawa, Poland; 3 Department of Ecology and Evolution, University of Lausanne, 1015 Biophore, Switzerland; 4 Max Planck Institute of Animal Behavior, Am Obstberg 1, 78315 Radolfzell, Germany; 5 Department of Biology, University of Konstanz, Universitätsstraße 10, 78457 Konstanz, Germany

**Keywords:** Ecophysiology, Reproductive biology, Metabolism

## Abstract

The ability of animals to produce endogenous heat provides a buffer against environmental changes but also incurs high energetic costs. Especially small endothermic mammals have high energy demands. Some temperate-zone species (heterotherms) regularly use torpor, which slows down their entire metabolism but also potentially delays reproduction, to compensate for this. We used a unique experimental approach to test the consequences of extended low and high ambient temperatures on the trade-off in energy allocation to body mass maintenance, thermoregulation effort and seasonal sexual maturation in temperate zone male bats. We showed that long exposure to low ambient temperature shifts energy allocation away from sexual maturation to self-maintenance and results in a delay of sperm maturation by as much as an entire month. This effect was partially buffered by higher body mass. Heavier bats were able to afford more intensive thermoregulation and consequently speed up maturation. Interestingly, bats at constant high temperatures avoided deep torpor and matured faster than those at low temperatures, but sperm production was also slower than under natural conditions. Our results show that not only low, but also constant high ambient temperatures are detrimental during seasonal sexual maturation and the trade-off between investing into self-maintenance and fitness is a finely tuned compromise.

## Introduction

Reproductive performance and survival are determined by fundamental energetic trade-offs. Animals are often faced with unpredictable environments due to variation in environmental conditions, such as food availability or weather, which makes balancing their energy budget challenging^1,2^. Many animals possess mechanisms to cope with such environmental variation. These mechanisms can be physiological (such as changes in metabolic rate, body temperature, or digestive function) or behavioural (including foraging behaviour, movement or roost choice)^3–5^. For example, endogenous heat production buffers the effects of variation in ambient temperature on important biological functions and reduces dependence on weather. Yet, endothermy is energetically demanding^6,7^. This demand is particularly high in small animals, including bats, as they have high metabolisms and lose heat rapidly due to their high surface to volume ratio^8^. Bats especially, are prone to heat and water loss due to the large naked surface of their wing membranes and the poor insulation properties of their pelage^9,10^.

Bats commonly compensate for energetic constraints by using torpor. Torpor is characterized by controlled metabolic suppression and lower body temperature^11,12^. Torpor reduces energy expenditure, thus allowing conservation of fat reserves^13^, which improves self-maintenance and survival during energetic bottlenecks^11,14,15^. Yet, torpor is also costly. A low metabolic rate down-regulates other physiological processes including those related to reproduction (e.g.,^16–18^). For example, testicle tissue becomes unresponsive to endocrine signals at low temperatures, and thus spermatogenesis proceeds at a slower pace^19,20^. In the large order of bats, the effects of torpor on reproduction have been studied almost exclusively in females (e.g.^17,21,22^) where time spent at low body temperatures negatively influences milk production and foetal development^17,22^. The effects of torpor on male reproductive performance remain poorly understood.

In bats from the temperate zone, sperm production and mating are temporally separated^23^. In spring, the testes grow in size due to enlargement of the tubular diameter^24,25^ and reach their maximum size during summer^25^. Afterwards, the spermatozoa are transferred to the *cauda epididymis* for storage until mating, and the testes regress again^1,25^. This process is repeated every year in mature males, which can be referred as “seasonal sexual maturation”^26^. Spermatogenesis is costly; due to intensive tissue growth of the seminiferous epithelium, testes can reach as much as 8% of body mass^27^. Moreover, the need for nutrients to proliferate germ cells increases (reviewed by Pescovitz et. al^28^ and Sharpe et. al^29^). At the same time, male bats should limit torpor use (e.g.^30^). Indeed, a reduced metabolic rate is suggested to slow down sexual maturation^31^. It was previously demonstrated that male bats exposed to both high and low food treatments all avoided torpor use during spermatogenesis^26^. However, males on a restricted diet reached full sexual maturation later than well-fed males because they were forced to allocate energy into maintaining their body mass, resulting in slower maturation. Thus, avoiding torpor is necessary to keep sperm production going, but may need to be balanced against survival^32^.

Several strategies for balancing energy allocation between thermoregulation and spermatogenesis in bats have been proposed. For example, bats can increase foraging activity and hence food intake^14,30,33,34^. Another suggested strategy to optimize energy budget during spermatogenesis is to become social. The males of some species of bats form temporary bachelor colonies during summer^35^, probably to benefit from more efficient foraging on ephemeral insects and or social thermoregulation^36^. Additionally, social thermoregulation reduces heat loss. Finally, if conditions are harsh, males may still use torpor (e.g.^26,36–38^) but only during the coldest time of the day, when the cost of heat production is the highest^36,37^. This compromise should promote survival, but at the cost of reduced or slowed-down sperm production^31,39,40^.

It is challenging to disentangle the effects of ambient temperature and food availability on the pace of sexual maturation in free ranging animals, but especially on insectivorous male bats. Insect activity, and thus food availability covary with weather conditions and affect bats' behaviour^36,41^, but the direct effect of temperature on sexual maturation remains untested. In this study, we experimentally tested the trade-off between energy allocated to thermoregulation and to seasonal sexual maturation in male parti-coloured bats, *Vespertilio murinus*. We exposed bats to two different ambient temperatures in order to mimic the upper and lower range of temperatures they may experience in their natural environment. The "warm" treatment temperature (25 °C) was close to the bats’ thermoneutral zone. The "cold" treatment (10 °C) was in the lower range of local temperature usually recorded during the corresponding time period (average minima and maxima for natural ambient temperatures in Białowieża in years 2014—2016: June : 6.9–27.9 °C, July: 6.6–32.4 °C, August: 5.05—32 °C; local weather stationin Białowieża). Additionally, we controlled for food intake to avoid complex confounding effects on sexual maturation rate^26^. *Vespertilio murinus* is one of the species where males aggregate in colonies during summer^35^. Colonies are formed during the time of spermatogenesis, and it has been hypothesized that colonial males profit from social thermoregulation, similar to reproductive females, to promote sperm maturation rate and to be ready for mating sooner^35,42^. Thus, this species presents an ideal system to test the trade-offs in energy allocation to self-maintenance versus reproduction.

There are two potential scenarios how low ambient temperature might affect seasonal sexual maturation rate in male bats. In scenario one, we hypothesized that low ambient temperatures should increase torpor use, but males in both treatments should avoid body temperatures close to ambient temperatures and actively thermoregulate in order to maintain sperm production. However, costs of thermoregulation should then be relevantly higher for individuals kept at low ambient temperature. As a result, to balance their energy budget, bats kept at low temperature may gain less or even lose mass, but sexual maturation would not be significantly delayed. Heavier individuals should have larger energy resources, facilitating investment into reproduction. In scenario two, we hypothesized that male bats should prioritize self-maintenance and body mass should not decrease. They should then use torpor and thermoconform to low ambient temperature at the expense of slowed-down or even suspended sexual maturation.

## Results

### Body mass

**Figure 1:**
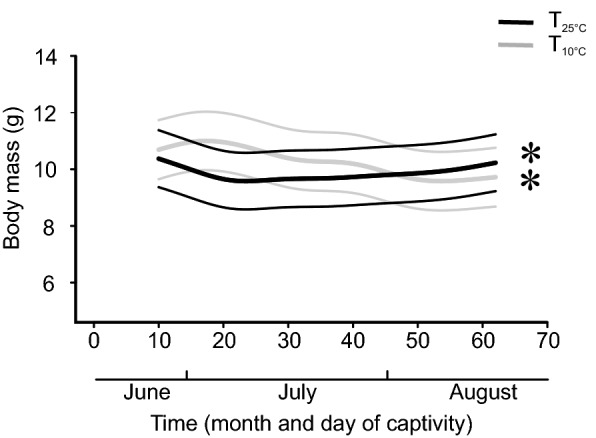
Modelled body mass of male bats in the two treatment groups. Data are shown in relation to time grouped by treatment. Thick lines represent the predicted body mass and thinner lines are 95% CI. Asterisks indicate significant effects.

The best model for body mass (M_*b*_) included forearm length (FA) as a proxy for size, time (day since capture) grouped by treatment, and treatment as independent variable (Table S1). There was no significant effect of FA or treatment itself on M_*b*_ (*P* > 0.05; Table 1). However, changes within each treatment over time were significant (T_25°C_ P < 0.001, T_10°C_ P < 0.001; Fig. 1, Table 1). In the T_25°C_ treatment, M_*b*_ of bats decreased during the first ten days of being exposed to 25 °C (Table 1) from an initial mean of 11.4 g (range: 10.1–12.6 g) to 10.2 g (range: 9.2–11.1 g; Fig. 1). After 20 days (ten days of experiment, in the beginning of July), we observed a small but constant increase of M_*b*_ (Fig. 1). The final M_*b*_ did not differ from the initial values (mean 11.0 g, range: 9.3–13.5 g). In contrast, mean M_*b*_ of bats in the T_10°C_ treatment increased during the first seven days of being kept at 10 °C (Table 1) from 11.7 g (range: 9.9–13.1 g) to 12.0 g (range: 9.7–13.9 g). After day 20, this trend switched to a constant decrease until the end of the experiment (mid-August). The final mean M_*b*_ was 10.7 g (range: 8.6–13.4 g) (Fig. 1, Table 1).

**Table 1:** Summary of GAMM model testing for M_*b*_.

Parameter	Estimate	SE	t value	*P*
Intercept	5.704	2.807	2.032	0.042
T_25°C_	− 0.418	0.385	− 1.088	0.277
FA	0.122	0.063	1.942	0.052

Nonparametric fit	edf	Ref.df	F	*P*
s(time): T_10°C_	7.044	8.116	115.630	< 0.001
s(time): T_25°C_	6.233	7.398	24.950	< 0.001
s(group)	4.342	10.000	9446.030	0.046
s(ID)	24.507	29.000	3931.060	0.009

Parameter estimates of general additive mixed model (GAMM) describing the effects of forearm length (FA), treatment (T_10°C_ and T_25°C_) and day since capture (time) on body mass (M_b_). Bat ID (ID) and group were included as random effects. Adjusted R^2^ = 0.851, scale estimates = 0.17776, n = 1455.

### Thermoregulation

The mean daily skin temperature (T_sk_) was different between treatments. In the T_25°C_ treatment the mean daily T_sk_ increased from an initial 28.1 °C (range: 25.5–30.7 °C) up to a mean 30.9 °C (range 29.0–32.0 °C) after 20 days of treatment and then slowly decreased to 28.2 °C (range 27.0–30.4 °C). In the T_10°C_ treatment the mean daily T_sk_ constantly decreased throughout the course of the experiment, from an initial 24.8 °C (range: 22.9–27.3 °C) down to 12.2 °C (range: 10–15.7 °C) at the end of the experiment. In the T_25°C_ treatment, skin temperature fluctuated during the day (with the range between 24.2 °C and 30.6 °C). The mean T_sk—onset_ (the threshold T_sk_ below which we considered bat to be in torpor) was 32.2 °C (range 31.7–32.7 °C, established for each individual separately). There was an average of 2.6 torpor events per day (range: 1–7) and mean torpor event duration was 14.7 h (range: 4.7–17.8 h). At T_10°C_ there was an average of one torpor event per day (range: 1–3) and mean torpor event duration was 17.6 h (range: 6.3–17.8 h) at a mean T_sk_ of 10.7 °C (range: 8.6–24.4 °C). Once bats decreased their T_sk_ to almost as low as T_a_ after evening handling, they remained torpid until evening arousal.

**Figure 2:**
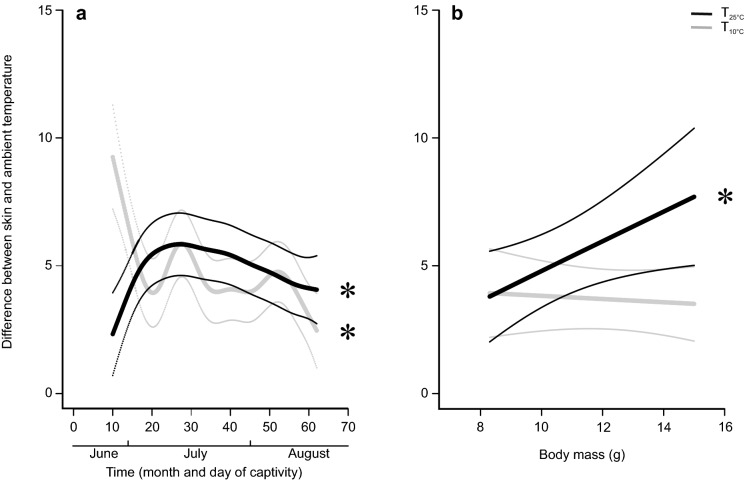
Modelled ΔT of male bats in the two treatments. Data are shown in relation to (**A**) time and (**B**) body mass (M_*b*_)both grouped by treatment (T_25°C_ in black and T_10°C_ in grey). Thinner lines represent 95% confidential intervals (CI). Asterisks indicate significant effects.

The best model for mean daily difference (ΔT) between ambient temperature (T_a_) and T_sk_, (i.e., estimated thermoregulation effort), included time grouped by treatment, M_*b*_ grouped by treatment and treatment as an independent variable (Table S2). There was no difference in ΔT between treatments (*P* > 0.05; Fig. 2A, Table 2). However, there were significant changes within each treatment over time (T_25°C_ *P* < 0.001, T_10°C_ *P* < 0.001; Table 2). After 18 days at T_25°C_, ΔT increased from 2.5 to 5.7 °C (Fig. 2A) and remained elevated but decreased slowly (Fig. 2A, Table 2). In contrast, T_10°C_ animals had an average ΔT of 8.6 °C at the beginning of treatment, which decreased within 10 days down to 3.9 °C. Later there were two peaks of ΔT: a stronger one around day 28 (5.8 °C) and a smaller one around day 52. (4.7 °C) (Fig. 2A, Table 2). M_*b*_ was significantly positively correlated with ΔT in the T_25°C_ treatment (*P* > 0.05; Fig. 2B, Table 2).

**Table 2:** Summary of GAMM model testing for ΔT.

Parameter	Estimate	SE	t value	*P*
Intercept	3.602	0.307	11.742	< 0.001
T_25°C_	0.635	0.440	1.444	0.15

Nonparametric fit	edf	Ref.df	F	*P*
s(time): T_10°C_	7.907	8.687	11.108	< 0.001
s(time): T_25°C_	5.-25	6.110	6.890	< 0.001
s(M_*b*_): T_10°C_	1.001	1.002	0.146	0.704
s(M_*b*_): T_25°C_	1.011	1.020	5.222	0.021
s(group)	4.422	10.000	8.691	0.195
s(ID)	12.240	21.000	5.423	0.064

Parameter estimates of general additive mixed model (GAMM) describing the effects of body mass (M_*b*_), consecutive day of observation (time) and treatment (T_10°C_ and T_25°C_) on difference between T_sk_ and T_a (_ΔT). Bat ID (ID) and group were included as random effects. Adjusted R^2^ = 0.33, scale estimates = 2.162, n = 5746.

**Figure 3:**
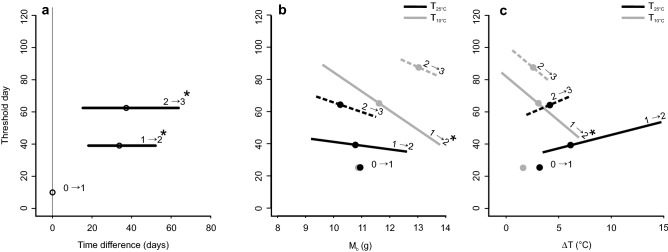
Sexual maturation rate of bats in the two treatments. The y-axis shows the average experimental day on which T_25°C_ bats reached the next consecutive maturation class. (**A**) Delay in time until T_10°C_ bats reached the same threshold. The y-axis shows the average day since the beginning of the experiment on which T_25 °C_ bats reached the next consecutive maturation class. The x-axis shows the delay in time until T_10 °C_ bats reached the same class. Each delay is presented with its 95% credible intervals (CI). (**B**) The effect of M_*b*_ (body mass) on the time of transition to consecutive classes. (**C**) The effect of ΔT (difference between T_sk_ and T_a_) on the transition time to consecutive classes. Asterisks indicate significant effects.

### Seasonal sexual maturation

Only one bat in the entire experiment reached seasonal sexual maturation class 4. This male was from the T_25°C_ treatment. Thus, posterior distributions of the effects related to the transition from sexual maturation class 3 to 4 represented the prior distributions rather than any effect calculated from the data. We thus did not consider the fourth threshold. At T_10°C_ only two males reached class 3.

Bats kept at T_25°C_ reached sexual maturation class 2 on day 39 (end of July) on average, while T_10°C_ bats were significantly delayed by 34 days on average (Table 3; Fig. 3A). The time needed to switch to class 3 was 62 days (counting from the beginning of the captivity) for T_25°C_ on average (mid-August). Bats from the T_10°C_ treatment required significantly more time: 37 days (end of September) (Table 3, Fig. 3A). At T_25°C_ there was no significant effect of M_*b*_ or ΔT on sexual maturation rate at any of the transition time points (Table 3). At T_10°C_ both M_*b*_ and ΔT had a significant effect only on the transition from class 1 to 2 (Table 3, Fig. 3B, C). The heavier an individual, the less time it needed to reach class 2 (Table 3, Fig. 3B). Similarly, the higher ΔT and thus thermoregulation effort, the faster the transition from sexual maturation class 1 to 2 (Table 3, Fig. 3C).

**Table 3:** Parameter estimates for seasonal sexual maturation rate.

A	Effect	Transition	Transition time for T_25°C_ treatment			
			Mean	Sd	50%	95% CI
	T_25°C_	1 → 2	39.37	4.72	39.07	31.08–49.49
		2 → 3	62.81	5.03	62.47	53.95–73.59

B	Effect	Transition	Delay in days			
			Mean	Sd	50%	95% CI
	T_10°C_	1 → 2	33.48	8.41	33.07	18.26–51.08 *
		2 → 3	37.12	12.3	36.35	14.86–63.34 *
	M_b_ T_25°C_	1 → 2	− 2.19	3.05	− 2.33	− 7.8–4.11
		2 → 3	− 5.89	3.77	− 5.93	− 13.16–1.58
	M_b_ T_10°C_	1 → 2	− 11.9	4.16	− 11.8	− 20.32 to − 4.00 *
		2 → 3	− 7.54	6.04	− 7.57	− 19.41–4.53
	ΔT T_25°C_	1 → 2	1.63	1.63	1.57	− 1.28–4.96
		2 → 3	2.72	2.16	2.56	− 1.07–7.39
	ΔT T_10°C_	1 → 2	− 5.38	2.46	− 5.5	− 9.93 to − 0.09 *
		2 → 3	− 5.32	3.42	− 5.35	− 12.1–1.7

(A) Transition time between sexual maturation classes for bats from the T_25°C_ treatment (in days since capture). (B) Effects of T_10°C_ and M_b_ and ΔT within each treatment expressed as delay in transition between seasonal sexual maturation classes. Posterior distributions are presented by their mean, standard deviation and by the 2.5th, 50th and 97.5th percentiles. Significant effects (i.e., 95% credible interval does not overlap 0) are marked with asterisks. Effects' unit is in day.

## Discussion

In nature, balancing the energetic trade-off between self-maintenance and reproduction can become challenging when animals face harsh environmental conditions. We investigated how heterothermic males prioritize energy allocation during seasonal sexual maturation.

As expected, we found that male bats exposed to T_25°C_ seasonally sexually matured faster and reached a more advanced stage than those kept at T_10°C_. Extrapolating from this results, bats from the T_10°C_ treatment would have reached sexual maturity almost a month later than those from T_25°C_ treatment. Sexual maturation rate is expected to depend on the energy balance between food intake, self-maintenance, and investment in thermoregulation^26^. We thus expected that heavier bats would have more energy to allocate to thermoregulation while still maintaining sperm production. We did find a significant effect of body mass and thermoregulation on seasonal sexual maturation rate, but only among bats exposed to low ambient temperatures. Above a certain level ambient and thus body temperature might be high enough to maintain a high rate of sperm production, but it is not possible to further speed up this physiological process without additional food^2^.

Despite being exposed to extremely different ambient temperatures, males did not show significant differences in body mass between the two treatments although weights of bats from the T_10°C_ treatment decreased slightly. This small but constant loss of weight indicates that food intake was not sufficient to balance the energy budget. Bats can deplete their fat reserves if are not able to balance their energy budget, in this case due to increased demands of sexual maturation^30,31,37^.

Individuals kept at low ambient temperature spent most of the time thermoconforming, i.e., their body temperature remained close to ambient temperature. For those individuals, the mean difference between body and ambient temperature was mainly a consequence of morning cooling and evening arousals. Morning cooling was a passive process and probably depended strongly on the bats’ digestion intensity. Nevertheless, even these short term increases in energy expenditures may have exceeded the energy intake of the bats and caused the observed weight loss. In contrast, bats from the T_25°C_ treatment actively thermoregulated almost continuously, even though they did not completely maintain normothermia either, and were in shallow torpor. This was a consequence of high ambient temperature, which made deeper torpor impossible. However, even shallow torpor can enable animals to save some energy^43^.

Partly in line with our prediction, we found a significant correlation between body mass and ΔT, but interestingly only in the T_25°C_ treatment. In this treatment, heavier individuals may have allocated energy to thermoregulation as well as spermatogenesis, while maintaining mass, similar to what was found in ad libitum fed bats in a food deprivation experiment^26^. Our results correspond to other observations^44,45^ which showed that torpor use in other small heterothermic mammals decreases with an increase in body mass.

Sexual maturation of individuals exposed to low ambient temperature was strongly slowed down. The short periods of raised metabolic rate and body temperature during feeding and digesting may have been sufficient to drive marginal testicular growth. Similarly, spermatogenesis of ground squirrels is likely restricted to arousal periods from torpor^19^, as spermatogenesis proceeds more slowly at lower body temperatures^20^. Heavier bats which likely had more fat reserves, may have been able to use the time of elevated body temperature to invest more in sexual maturation, despite similar thermoregulation effort in all "cold" bats.

Decreased thermoregulation effort and higher body mass significantly reduced the time for transition from maturation class 1 to 2 (at T_10°C_). During the seasonal sexual maturation process, this particular stage corresponds to intense production of gametes (reviewed by ^25,46^). The seminiferous epithelium of the testis is sensitive to cooling^47^. In mammals including heterothermic species, such as hedgehogs^48^ the enzyme system in the testes works most efficiently at a very narrow temperature range^49,50^. Mammalian cell growth and gonadal steroidogenesis are generally strongly reduced at body temperatures below 20 °C (e.g.^49,50^). Consequently, deep and prolonged torpor may cause stagnation of spermatogenesis at various stages of meiosis^19,20^. Our results support the assumption that some heterothermic species appear to have mechanisms which enable at least partial continued gametogenesis and endocrine responsiveness at low temperatures^19^. Low temperatures are favourable for sperm storage^51^, which suggests that later thermoregulation can be relaxed during the transfer of spermatozoa to the *cauda epididymis*.

Free-ranging colonies of male *V. murinus* break up after reaching sexual maturation stage 2^36,42,52,53^. After this stage, according to our results, the need for maintaining high body temperature is lessened. Our results thus provide evidence to support the social warming hypothesis^35,42^ explaining why male bats form colonies.

**Figure 4:**
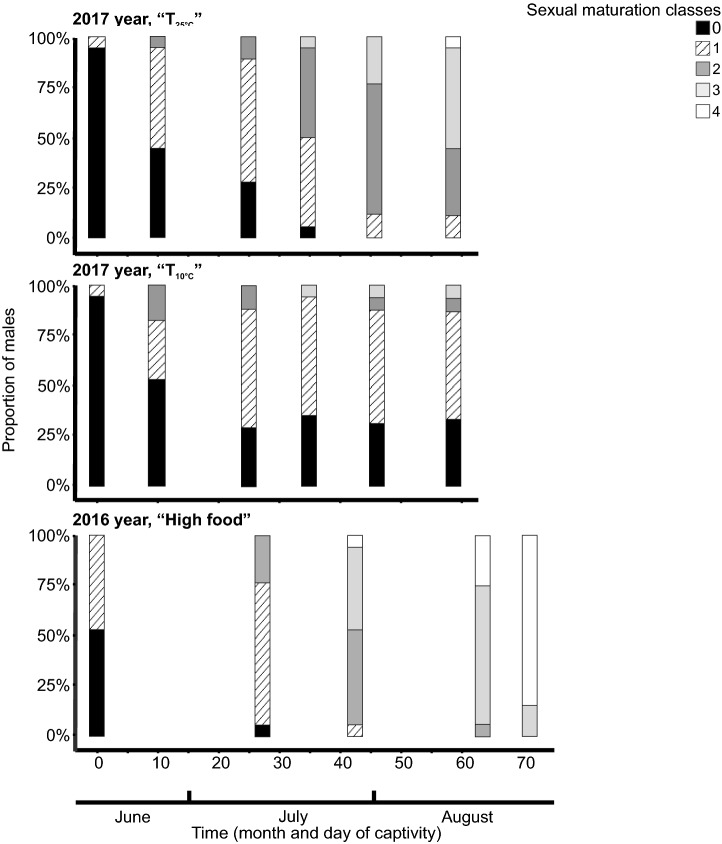
Effect of high and low ambient temperature or food availability on seasonal sexual maturation rate. The proportion of male bats at each sexual maturation classes over the course of the study and under different treatments. Data from the “High food” treatment are from an experiment, where *V. murinus* males were fed with a large amount of food and exposed to natural ambient temperature^26^. Both experiments started on the same calendar days and the numbering of experimental days is the same in both studies.

We extrapolated from our results that bats from the T_25°C_ treatment would not reach sexual maturity until late September, if continued to be kept at 25 °C. Thus, bats exposed to constant elevated temperature would reach sexual maturity more than one month after wild individuals^36,53^ or captive ones maintained under naturally fluctuating ambient temperatures and provided with high amounts of food^26^ (Fig. 4). This coincides with the delay in food-restricted males^26^. Our results can suggest that at least brief periods of torpor use may favour in process of seasonal sexual maturation. This might be a result of the need for small energy savings to maintain spermatogenesis (see also above) or limiting water loss^11,43^. During periods of increased energy requirements, such as spermatogenesis, fine temperature tuning may be thus required to facilitate physiological processes. In nature bats can manipulate their body temperature in part by choosing suitable roosts according to their current needs^54,55^. However the role of brief periods of torpor in the process of seasonal sexual maturation requires more detailed studies.

Mating readiness and synchronisation with female receptivity should be under strong selective pressure in promiscuous species^56^. Males that encounter low ambient temperatures or abnormally high temperatures may not be ready for mating in a given year, which would have serious consequences for their reproductive success. Spermatogenesis takes places in spring shortly after hibernation in heterothermic species (e.g., chipmunks *Tamias striatus* and ground squirrels) which have higher reproductive success if they use less torpor during this period^19,57^. This delay in readiness for reproduction might be seen as a non-energetic cost of hibernation^58^ but also heterothermy in general. Male sociality during critical stages of seasonal sexual maturation has likely evolved to facilitate sperm production in species relying on ephemeral food resources.

## Methods

### Ethical statement

All experimental procedures were approved by the General Director for Environmental Protection in Poland (authorization no. DZPWg.6401.09.2.2014, DZP-Wg.6401.09.1.2015, DZPWg.6401.09.5.2016) and by the Local Ethical Commissions in Białystok and Olsztyn (authorization no. 11/2014, 14/2015, 120/2015, 150/2015, 15/ 2015, 45/2015). All methods were carried out in accordance with relevant guidelines and regulations. The study was performed in accordance with the ARRIVE guidelines.

### Experimental animals and housing conditions

We captured 36 bats from a free-ranging colony of approximately 100 males of *Vespertilio murinus* in mid-June 2017 in the Bieszczady region (SE Poland, 49°10′36″N 22°26′01″E, 644 m.a.s.l.). We caught bats during their evening emergence from the roost using a harp trap (Austbat, Bat Conservation and Management) and a custom-built funnel trap. We marked all individuals with subcutaneous PIT Tags (Trovan ID-100B /1.4, 8 mm), weighed them (± 0.1 g; Pesola PTS3000 General Electronic Scale) and measured their forearm length (± 0.1 cm) as a proxy for body size. Body mass (M_*b*_) at capture ranged from 9.4—12.8 g (mean 11.1 g) and forearm length (FA) from 34.0—46.9 mm (mean 44.0 mm). Bats were transported to the Mammal Research Institute of the Polish Academy of Sciences in Białowieża (MRI PAS), where the experiment took place. During an initial 10-day acclimation period, we trained bats to feed independently on mealworms (2 g/day each throughout the captive period based on previous experience^26^). This diet was supplemented with vitamins (solBiosupervit, Biofaktor, Skierniewice, Poland) added to the water (offered *at libitum*) every fourth day^26,59^. During the acclimation period, bats were housed in a closed flight room^26^. Bats were provided with hollow tree-trunks as roosts^60^ and were allowed to freely choose group size and composition. During the acclimation time, bats were allowed to fly freely for three hours every day. Upon completion of the experiment, we released the bats in the evening at the capture site. We defined the entire time when bats were in MRI PAS as “captivity” and as “experiment” when they were under temperature treatment ten days into captivity. We numbered days consecutively starting from the day of capture.

### Experimental design

Our experiment started at the end of June and lasted until mid-August (52 days), and thus overlapped with the social and solitary periods of *V. murinus*. Indeed, in eastern Poland, male *V. murinus* briefly form colonies from the beginning of June until the beginning of July^36,53^. We caught the males when they were at the early stage of testicle growth and kept them until seasonal sexual maturation should be strongly advanced if not completed^53^.

We exposed bats to two experimental temperature treatments. Half of the bats were kept at 10 °C (T_10°C_) and the others at 25 °C (T_25°C_). The 10 °C treatment was chosen as this is the lowest temperature usually recorded in the region during the experimental period. We chose 25 °C as the high temperature treatment to keep the bats below their thermoneutral zone^9^. Bats were assigned to treatments according to individual body condition index (FA / M_b_) at capture, resulting in two groups with the same BCI distribution. Due to health problems we had to exclude four individuals from the experiment (one from the T_25°C_ and three from the T_10°C_ treatment; final sample size: T_25˚C_ = 17; T_10˚C_ = 15). During the experiment bats from each treatment were kept in groups of three, in wire-mesh cages in temperature-controlled cabinets (Pol-Eko Aparatura, model ST 700 BASIC). This group size was chosen to avoid social isolation stress, while limiting social thermoregulation. Composition of each group remained the same throughout the experiment. We suspended folded paper towels in each cage to offer bats the opportunity to hide. We took bats out of the cages every evening for feeding and weighing. During the experiment, each group was offered the opportunity to fly for three hours every second day in the enclosure where they were kept during the acclimation period.

### Body mass and skin temperature

We measured M_*b*_ every evening before feeding. We assumed that the increase or decrease in M_*b*_ was related to changes in stored fat, and consequently to changes in body condition.

We measured skin temperature (T_sk_) as a proxy for body temperature using temperature data logger (iButtons, model DS1922L, Dallas Semiconductors, TX, US; miniaturized^61^) glued to the skin on the bats' back with tissue adhesive (Sauer Hautkleber, Manfred Sauer, Germany). iButtons weighed approximately 1.6 g (11–18% of individual M_*b*_), but did not disturb the animals or affect their ability to fly, based on our observations and experience^26^. iButtons recorded temperature every 10 min with a 0.5 °C-resolution. The iButtons detached spontaneously after some time, and duration of attachment varied from two weeks to two months. Once an iButton detached, we replaced it only after a break of two weeks minimum. Throughout the experiment at least one-third of the experimental animals (12 individuals, six from each treatment) were equipped with temperature loggers at the same time. We excluded T_sk_ recordings from time periods when bats were removed from the cage for everyday feeding and when they were flying.

We describe variation in thermoregulation effort using the mean daily difference (ΔT) between ambient temperature (T_a_) and T_sk_. ΔT allows us to directly compare energy expenditures, as an increase in body temperature (measured here as T_sk_) by one degree is expected to require the same energy input at any T_a_ below the thermoneutral zone^12,62^. We also described torpor use by the bats. We determined the torpor onset threshold for each individual following the Eq. ^63^:

$${\text{T}}_{{{\text{sk}} - {\text{onset}}}} - { }1{\text{SE}} = \left( {0.041} \right){\text{M}}b + \left( {0.04} \right){\text{T}}_{{\text{a}}} + 31.083$$

We assumed a bat was using torpor if at least three T_sk_ recordings in a row were below the threshold.

### Sexual maturation

We used a five-step classification based on testicular growth and epididymal filling to describe seasonal sexual maturation stages^52^. A single observer (EK) assessed stages through external visual examination of the testes and *caudae epididymis* approximately every ten days. Testicular maturation stages were assigned to five classes from 0 (no to small testes at the penis base with no *cauda epididymis* visible), through 2 (testes at their maximum size, situated below the penis and *cauda epididymis* clearly visible as dark patches, but still flat) up to 4 (testes regressed and *cauda epididymis* completely filled); classes 1 and 3 were intermediate between these classes^52^ (detailed description in supplementary materials^26^). Partial filling of *cauda epididymis* indicates high advancement in spermatogenesis and that spermatozoa are being moved from the testes to *cauda epididymis* for maturation^24,64^. We defined the time needed to move from one class to the next as sperm production rate (seasonal sexual maturation rate) and modelled this rate for each individual (see below).

### Statistics

#### Body mass and thermoregulation

We tested for the effects of the two temperature treatments on M_*b*_ and ΔT using Generalized Additive Mixed Models (GAMM, mgcv’ package^65^). Models testing for M_*b*_ included three explanatory variables: treatment, FA and time (day since capture). Models for the analysis of ΔT included time, M_*b*_ and treatment. In models testing for M_*b*_ and ΔT, all variables were continuous except for treatment which was a two-level factor (T_25°C_ and T_10°C_). We included bat ID nested in group as a random effect. We assumed that the differences in the effect of each factor on M_*b*_ and ΔT between treatments were significant when confidence intervals (CI) of treatments did not overlap. We selected the best models for M_*b*_ and ΔT based on Akaike’s information criterion (AIC, model selection in Table S1).

#### Seasonal sexual maturation rate

We estimated the effect of treatments as well as the interaction of M_*b*_ and ΔT with treatments, on seasonal sexual maturation over the course of the experiment. We used hierarchical ordinal models using Bayesian inferences^66^. M_b_ and ΔT corresponded to values recorded on the day of testes examination (expressed as sexual maturation class). Missing values in ΔT or M_*b*_ were modelled from prior Gaussian distributions characterised by single estimated means and precisions. Bats from the T_25°C_ treatment were considered as the baseline. We centred values of ΔT and M_*b*_. Consecutive days since capture were divided by 365 in order to ease the initial convergence of the chains.

A continuous latent sexual maturation variable was modelled and related to the time since capture with a random intercept linear mixed model. Bat ID was included as a random effect. We estimated the days corresponding to switches from one sexual maturation class to the next along the latent variable (defined as “transition time point”). We assumed that bats switched from class 0 to 1 independently from treatments, ΔT or M_*b*_. This class threshold was set at day 10 of captivity (i.e., the onset of the experiment). On that day, half of the bats had already passed the first threshold. We also estimated the effects of treatment, M_*b*_, ΔT and the interactions (treatments * M_*b*_ and treatments * ΔT) on the threshold between sexual maturation classes. Effects were considered significant when their posterior distribution 95% credible intervals (CI) did not overlap with 0.

We ran three different Markov chains starting at random initial values in the range of parameter space for 50′000 iterations with a 20′000-iteration burn-in. Markov chains were thinned by a factor of three and the Brooks-Gelman-Rubin criterion (R̂) was used to assess the convergence of chains, indicated when R̂ < 1.1^67^. We used the function ‘jags’ in the package: ‘jagsUI’^68^ to run the analysis. The model with the description of prior distributions can be found in the supplementary material.

## Supplementary Information

Supplementary Information.

## Data Availability

Data available from the Open Forest Data Repository: https://doi.org/10.48370/OFD/GEGGMQ.
