# Supplementary material for: Energy allocation shifts from sperm production to self-maintenance at low temperatures in male bats
Source: Sci Rep. 2022 Feb 8;12:2138. doi: 10.1038/s41598-022-05896-3 (PMC8826387; doi:10.1038/s41598-022-05896-3)
Supplement: Supplementary file 1 — Supplementary Information. [file 41598_2022_5896_MOESM1_ESM.pdf]

SUPPLEMENTARY MATERIALS

This document contains supplementary for methods and results for the following article:

Title: Energy allocation shifts from sperm production to self-maintenance at low temperatures in male bats

R script for seasonal sexual maturation modelling

```
sink("Testes.jags")
cat("
model {

for(i in 1:N){
                ## loop over observations
          ## form the linear predictor

                  mu[i] <-x[i]*beta+eps[id[i]]

           ## cumulative logistic probabilities
           logit(Q[i,1]) <- tau[1]-mu[i]
           p[i,1] <- Q[i,1]

           for(j in 2:4){
         logit(Q[i,j]) <-
         tau[j]+
         eff_T[j-1]*treat[i]+
         (int_deltaT[j-1]*treat[i]+eff_deltaT[j-1])*deltaT[i]+
         (int_BM[j-1]*treat[i]+eff_BM[j-1])*BM[i]+
         -mu[i]

         p[i,j] <- Q[i,j] - Q[i,j-1]
           }
           p[i,5] <- 1 - Q[i,4]
           y[i] ~ dcat(p[i,1:5])
           ## p[i,] sums to 1 for each i

           #priors for variables with NA
           deltaT[i]~dnorm(mu.deltaT,deltaT.prec)
           BM[i]~dnorm(mu.BM,BM.prec)
          }

## priors

           beta~ dnorm(0,0.001)
           mu.deltaT~ dnorm(0,0.001)
           mu.BM~ dnorm(0,0.001)
           deltaT.prec <- 1/pow(deltaT.sd,2)
           deltaT.sd~dunif(0,5)
           BM.prec <- 1/pow(BM.sd,2)
```

```
		BM.sd~dunif(0,5)

## hierarchical model over BAT_ID

for(k in 1: NID){
		eps[k] ~ dnorm(0,eta)}
	eta <- 1/pow(sd,2)
	sd ~ dunif(0,3)

## priors over thresholds constraint to be ordered

	for(i in 1:3){
	tau0[i]~dunif(tau[1],100)}

	tau[2:4] <- sort(tau0)
	tau[1] <- tauJ*beta

## priors over effects on thresholds

for(i in 1:3){
			eff_T[i] ~dnorm(0,0.001)
			eff_deltaT[i]~dnorm(0,0.001)
			int_deltaT[i]~dnorm(0,0.001)
			int_BM[i]~dnorm(0,0.001)
			eff_BM[i]~dnorm(0,0.001)}


}",fill=T)
sink()


# Bundle data
jags.data <- list(NID=NID,
		x=data$SESSION/365,
		y= data$TESTES,
		id=id,
		N=N,
		tauJ= tauJ,
		treat=data$REGIME,
		deltaT=data$T_diff-mean(data$T_diff,na.rm=T),
		BM=data$BM-mean(data$BM,na.rm=T))
#Initial values
inits <- function(){list(
	tau0 = runif(3,tauJ,1))}


# Parameters monitored
parameters <- c("beta","tau","eff_T","eff_deltaT","int_deltaT","eff_BM","int_BM","eps")

# MCMC settings
ni <- 50000
nt <- 3
```

```
nb <- 20000
nc <- 3

# Call JAGS from R
mtestes3<- jagsUI::jags(jags.data,
                  inits,
                  parameters,
                  paste("Testes.jags"),
                  n.chains = nc,
                  n.thin = nt,
                  n.iter = ni,
                  n.burnin = nb,
                  parallel=T)
```

Results

Table S1. Results of model selection (based on AIC criteria) for body mass fitted by general additive mixed models (GAMM). Variables: FA-forearm, time - consecutive day day since capture, Trt – treatment ($T_{10°C}$ and $T_{25°C}$). ID – bat ID and group – fixed groups in which bats were kept included as random effects. Headings for columns are: AIC (AIC), difference in AIC related to the optimal model (Δ*AICc*), number of parameters (K), percentage of deviance explained by the fitted model (Devexp).

Rank	*Covariates*	*AICc*	Δ*AICc*	K	*Devexp*
1	s(time, by=Trt)+FA+Trt	1524.304	0	5	86.3
2	s(time)+FA+Trt	2102.231	577.927	5	79.1
3		2325.991	801.687	3	75.1

Table S2. Results of model selection (based on AIC criteria) for the mean daily difference between T_b and T_{sk} (ΔT) fitted by general additive mixed models (GAMM). Variables: M_b - body mass, time- consecutive day day since capture, Trt – treatment ($T_{10°C}$ and $T_{25°C}$). ID – bat ID and group – fixed groups in which bats were kept included as random effects. Headings for columns are: AIC (AIC), difference in AIC related to the optimal model (Δ*AICc*), number of parameters (K), percentage of deviance explained by the fitted model (Devexp).

Rank	*Covariates*	*AICc*	Δ*AICc*	K	*Devexp*
1	s(time, by=Trt)+s(M_b, by=Trt)+Trt	2013.569	0	5	37
2	s(time, by=Trt)+s(M_b)+Trt	2015.872	2.303	5	37.2
3	s(time)+s(M_b, by=Trt)+Trt	2088.456	74.887	5	27.4
4	s(time)+s(M_b)+Trt	2099.798	86.229	5	24.2
5		2638.453	624.884	2	13.9
